# Report from MDE practice: An interview-based evaluation of model-driven engineering uses

**DOI:** 10.1371/journal.pone.0335461

**Published:** 2025-11-05

**Authors:** Hessa Alfraihi, Kevin Lano

**Affiliations:** 1 Department of Information Systems, College of Computer and Information Sciences, Princess Nourah bint Abdulrahman University, Riyadh, Saudi Arabia; 2 Department of Informatics, King’s College London, Strand, London, United Kingdom; Hanshan Normal University, CHINA

## Abstract

In this study, we investigate the usability of Model-Driven Engineering (MDE) through interviews with fifteen practitioners from diverse roles (e.g., developers, researchers, architects) and domains, and with a range of expertise levels across academic and industrial software sectors, capturing in-depth perspectives on its practical application. Participants emphasized MDE’s benefits in enhancing project robustness, reliability, development speed, and system organization. However, they also identified challenges such as a steep learning curve, technological constraints, organizational resistance, and a shortage of skilled professionals. To address these issues, participants recommended simplifying tools and language, improving consistency and flexibility, enhancing integration with existing workflows, and raising awareness of MDE. These insights provide valuable guidance for improving MDE usability and encouraging broader MDE adoption.

## 1 Introduction

Usability is a paramount consideration in any software engineering methodology, and Model-Driven Engineering (MDE) is no exception. While MDE has been recognized for its potential to enhance efficiency, productivity, quality, and maintainability of software systems, there is a growing consensus that its usability aspects require more rigorous examination. This study explores the practical usability of MDE, focusing on insights from fifteen practitioners in academia and industry.

In the context of MDE, usability, as defined in ISO 9241-11:2018 (Ergonomics of human-system interaction – Part 11: Usability: Definitions and concepts, International Organization for Standardization, 2018), refers to the effectiveness, efficiency, and satisfaction users derive from interacting with MDE tools, notations, and processes. In addition, it encompasses factors such as learnability, error management, cognitive load, and integration challenges. Even minor usability issues can hinder the productivity gains MDE aims to deliver.

To investigate these issues, we conducted semi-structured interviews with practitioners actively using MDE. The study aimed to identify strengths and weaknesses of current MDE practices and to gather recommendations for improving usability in real-world settings.

Analysis revealed recurring themes such as challenges of integrating MDE tools into workflows, steep learning curves, and cognitive load. Participants emphasized the need for better error management and more intuitive tools. These insights highlight the gap between MDE’s theoretical benefits and practical use and provide a basis for targeted usability improvements.

Examining usability from a practitioner’s perspective sheds light on gaps between theory and practice. The findings contribute to a holistic understanding of MDE and identify areas vital for enhancing adoption and effective implementation. This study fills a critical gap in MDE research by offering a ground-up examination informed by real user experiences.

The remainder of this paper is structured as follows: Sect 2 covers the background on Model-Driven Engineering (MDE), including key concepts, theoretical benefits, and related literature on usability. Sect 3 outlines the research methodology, including the semi-structured interviews, participant selection, and data analysis. Sect 4 presents the study’s results, focusing on key themes like MDE strengths, weaknesses, and usability challenges. Sect 5 discusses these findings in relation to existing literature and their implications for MDE adoption. Sect 6 addresses the study’s limitations. Sect 7 compares our findings with related work, and Sect 8 concludes with key insights, recommendations, and future research directions.

## 2 Background

In recent years the importance of usability and user experience (UX) in the application of MDE has been increasingly recognised. The study of [1] identified the need to evaluate and improve the UX of MDE tools to reduce the cognitive load of MDE application. The survey of [2] also identified the complexity of graphical modelling tools as a significant obstacle to MDE adoption, and recommended the increased use of textual modelling.

Our team conducted a preliminary study of MDE application, by means of an online survey with 119 participants from academia and industry: [3]. The survey explored the broad aspects of MDE usability, identifying key trends and challenges. The insights gained from the survey pointed towards the need for a deeper and more nuanced understanding of MDE usability. While the survey of [3] provided valuable high-level insights, it also revealed a rich complexity of opinions and experiences that warranted further investigation. A more detailed exploration through one-on-one interviews was therefore considered essential in order to fully comprehend the multifaceted nature of MDE application in practice. Thus, the present empirical study seeks to complement the preliminary survey by investigating in depth the complex details, positive and negative aspects, and practitioners’ recommendations for changes and improvements in MDE. Specifically, this study focuses on understanding the positive and negative aspects of MDE, exploring recommendations for changes and improvements, in order to complement and enrich the preliminary survey findings.

The purpose of this research is to uncover detailed insights that could not be captured through an online survey alone. The findings are intended to contribute to a more comprehensive and actionable understanding of MDE, fostering a pathway towards improvements and innovations in the field.

## 3 Methodology

This study uses semi-structured interviews and subsequent qualitative content analysis as the key research methodology. This approach aligns with the guidelines detailed by [4] and [5]. Semi-structured interviews were selected as the data collection method because of their versatility and effectiveness in capturing expert perspectives and insights. They offer a robust framework that allows researchers to explore the knowledge, thoughts, and opinions of experts in the field of Model-Driven Engineering (MDE), both from academia and industry. The research methodology was comprised of four primary stages, which we will detail further in the corresponding subsections:

1. **Preparation**: Initially, we clarified the research objectives and formulated the research questions that would guide our investigation (Sect 3.1). The methodological framework was established, including the selection criteria for interviewees and the draft structure for the interviews (Sects 3.2, 3.2.2, and 3.2.3 respectively). We also fine-tuned our questions, in order to investigate and gain further insights into the usability of MDE.
2. **Conduction of Interviews**: Using a semi-structured approach, we conducted interviews with fifteen professionals in the field (Sect 3.3).
3. **Data Analysis**: To extract meaningful patterns and themes from the collected data, we employed a three-tiered coding method (Sect 3.4). This allowed for an in-depth understanding of the participants’ perspectives and experiences.
4. **Interpretation and Synthesis** Upon completion of the data analysis, we interpreted the findings and synthesized them into a cohesive narrative that presents an overview of the usability of MDE from the viewpoint of our interviewees (Sect 4).

Each step of the process was carefully designed and executed to ensure that our findings were both reliable and valuable to the ongoing discourse on MDE usability. We believe that our results provide crucial insights that could inform future research and practice in this field.

### 3.1 Research objective and research questions

The objective of this research is to investigate the effectiveness and usability of Model-Driven Engineering (MDE) tools, methods, and notations in practical applications. The research aims to identify the MDE tools and methods that work well and those that work poorly in practice, as well as to propose potential changes and improvements to enhance MDE tool and method effectiveness. In order to achieve our objective, we formulated the following questions:

**Q1.** How effective have various MDE tools/methods/notations been in practice?
  **Q1.1.** What are the main strengths of MDE as a discipline and in terms of the tools and methods available?
  **Q1.2.** What are the main weaknesses or limitations of MDE tools or methods?
**Q2.** What changes to MDE tools/methods/notations could improve its effectiveness?
  **Q2.1.** What are the most important features that should be present in MDE tools, methods, or notations to enhance their effectiveness?
  **Q2.2.** What specific changes or additions are recommended to increase the effectiveness of current MDE tools, methods, or notations?

### 3.2 Study design

In order to investigate in depth the usability aspects of MDE, we carried out a study involving 15 practitioners actively engaged in MDE. Our research approach involved conducting online semi-structured interviews, enabling us to gather insightful perspectives from these experts. By employing open-ended questions, we aimed to foster a comprehensive exploration of the topic, allowing the participants to express their thoughts and experiences freely.

#### 3.2.1 Study type selection.

To obtain a comprehensive understanding of the field, we decided to conduct an interview study. Following the recommendation by [6], we specifically chose semi-structured interviews as these allow for both descriptive and explanatory exploration of the subject matter. Compared to a survey study, conducting interviews offers numerous advantages. Firstly, it minimizes the risk of misunderstandings that can arise in written surveys. Furthermore, interviews are more likely to yield comprehensive responses to open-ended questions, striking a balance between detailed and broad perspectives. Although the interview study resulted in a smaller number of responses, compared to our survey of [3], the participants offered more profound and insightful observations compared to a survey study. Additionally, prior to the interviews, we took care to ensure that the participants were aligned with our target group, to establish the relevance of their participation and expertise for our study.

#### 3.2.2 Participant selection.

Participants were initially selected from those respondents to our previous survey [3] who had expressed interest in further engagement. Of these, eight agreed to participate in follow-up interviews. Additionally, we reached out to 20 individuals identified from their studies and involvement in MDE through purposive sampling, inviting them to contribute their insights. These individuals were chosen for their expertise in both academic and industrial contexts, aiming to ensure a broad representation of perspectives. Of the 20, seven agreed to participate. This process allowed us to capture a wide range of experiences across different roles and settings. In total, 15 practitioners from both research and industry participated in the study. While the sample was not randomly selected, it was designed to ensure relevance and depth of expertise. A more detailed discussion of the methodological limitations, including issues of generalizability, is provided in the Limitations section.

#### 3.2.3 Interview structure.

Our interviews were semi-structured, offering flexibility to explore different facets of the participants’ experiences with MDE, while maintaining a consistent focus on our core research areas. At the beginning of each interview, a clear, common definition of Model-Driven Engineering (MDE) was provided to ensure that all participants had a shared understanding of the concept in the context of the study. This definition emphasized MDE as a software development methodology that focuses on creating and exploiting domain models as the primary means of information exchange between engineers, allowing for abstraction, automation, and improved efficiency in software development processes.

The key topics covered in the interviews included the participants’ experiences with MDE, their perceived benefits and challenges, the extent of MDE adoption within their professional or academic setting, and their perspective on the future of MDE. The interview guide is presented in S1 Appendix. The interview questions were designed to understand the positive aspects and strengths of MDE, both as a discipline and in terms of practical application (Q1.1). Likewise, they sought to identify the challenges and limitations associated with MDE tools and methods (Q1.2). In addition, interview questions focused on eliciting suggestions for enhancing the effectiveness of MDE tools and methods by identifying crucial features that participants believe should be incorporated (Q2.1). Participants were also asked to provide specific recommendations for improving existing MDE tools, methods, or notations, offering practical insights into potential enhancements that could address the identified weaknesses and limitations of current practices (Q2.2).

### 3.3 Data collection

The interviews were conducted remotely between March and December 2023, primarily due to geographic considerations. Prior to the interviews, participants were sent an information and consent letter outlining the study objectives, procedures, and their rights. Verbal consent for participation and audio recording was then obtained at the start of each interview. This verbal consent process was audio recorded as part of the interview session. The complete information and consent form can be found in S2 Appendix.

The assembled body of data comprises approximately 12 hours of recorded interviews and extends to over 110,000 words of transcribed content. Together, our group of interviewees have amassed over 260 years of combined experience in MDE, drawn from many roles within diverse organizational structures and from various application domains ranging from AI and robotics to finance and telecoms. In our analysis, we applied a grounded approach, enabling us to identify emerging themes and issues from the data. We have sought to understand not only what these themes are, but also the multifaceted ways in which they are articulated and manifested in the discourse of our study participants. This comprehensive approach ensures a rich exploration of the data, helping to bring out the nuances and depths of the subjects at hand.

### 3.4 Data analysis

We conducted a thematic analysis following the six-phase framework proposed by Braun and Clarke [7]. This approach was selected for its flexibility and suitability for capturing rich, qualitative insights from semi-structured interviews. It is a thematic analysis, which involves identifying, analysing, and reporting patterns (themes) within qualitative data to provide a rich, detailed account of the dataset. After transcribing the interviews verbatim, the first author conducted an initial review to become familiar with the data. Using an Excel spreadsheet, the first author then carried out systematic coding, identifying meaningful segments and organizing them into preliminary codes. These codes were then grouped into potential sub-themes and broader themes.

**Fig 1:**
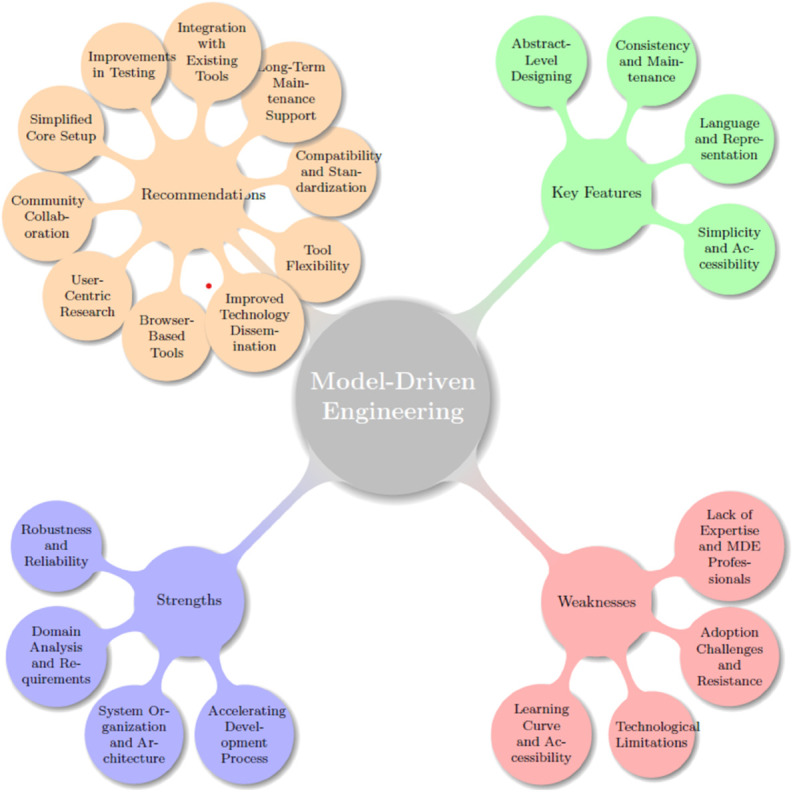
Themes and subthemes of MDE effectiveness and improvement.

Although the primary coding and theme development were conducted by the first author, the second author was actively involved in reviewing and discussing the evolving themes. Through ongoing conversations, both authors collaboratively interpreted the data, refined the themes, and ensured alignment with the research questions. While we did not apply formal inter-rater reliability measures or use a predefined codebook, this iterative process of collaborative reflection served as an informal validation approach. It supported the credibility and consistency of the analysis while allowing flexibility in interpreting complex qualitative insights. A visual summary of the final themes and sub-themes is presented in Fig 1.

### 3.5 Validity procedures

In an effort to ensure the validity of our findings and to diminish potential risks, we put in place a series of rigorous protocols. Our research process was designed with careful review procedures integral to each step. The first author took responsibility for conducting the interviews and performing the initial analysis, while the co-author systematically reviewed each stage of the process.

Whenever ambiguity arose in our interpretation of specific statements or outcomes, we addressed this by engaging in thorough discussions among ourselves to align our understandings, or we sought further clarification from the interviewees. This method was instrumental in ensuring a clear and consistent understanding of the findings and mitigating any risk of misinterpretation.

To mitigate any implicit assumptions on our part during the analysis and to minimize bias, we randomized the order of interview analysis, ensuring that it varied from the sequence in which the interviews were conducted.

## 4 Results

This section presents the results of the interview-based research, where 15 participants, each having a main role in a specific Model-Driven Engineering (MDE) project, reflect on their experiences. Each participant was asked to elaborate on their project, including descriptions, the development team’s constitution, their main role, the project’s duration, scale, and domain, among other aspects.

### 4.1 Participant demographics

Our group of 15 interviewees brings a wide range of experiences from both academia and industry, providing a diverse set of perspectives. Five of the participants are from a primarily academic background, two of whom also have experience working in an industrial context. This academic grounding should provide valuable insights from a more theoretical and research-driven viewpoint. Six participants have experience in both academia and industry, providing insights from both theoretical and practical realms. Lastly, we have four participants who are employed in the software industry, from companies in the health, defense, and finance sectors. Their experience provides practical knowledge of real-world MDE applications. Collectively, this group’s varied background ensures a broad spectrum of viewpoints, enhancing the depth and breadth of our inquiry.

Regarding the work experience in terms of years, the interviewees have a wide range of experience from three years to more than 30 years in MDE. One participant is just beginning their journey with three years of work experience, while at the other end of the spectrum, there are MDE veterans with over three decades of engagement.

Lastly, with regard to application domains, there is an equally diverse range of areas. The areas covered include AI recommender systems, MDE tools, defense systems, mobile apps for the medical domain, robotics, finance and insurance, data science, low-code development, banking, cyber-physical systems, safety-critical domains, automotive industry, embedded systems, and telecommunications. Table 1 presents the demographic information of participants.

### 4.2 Project descriptions

At the beginning of the interview, the participants were asked to choose a specific project and reflect on their experience of applying MDE in this project. By focusing on a specific project, the participants were able to provide detailed and specific information about their experiences with MDE. The goal of this question was to gain a deeper understanding of how MDE was applied, its effectiveness in that particular context, and any challenges or benefits observed. Each participant provided a detailed description of their respective MDE project, including its application domain, scale, development team size, duration, and the overall context in which the project was executed. Table 2 provides a summary of project descriptions. The projects for each participant are as follows:

- **Project 1: AI-Based Recommender System for Model and Metamodel Construction**
  This project aimed to develop an AI-based Recommender System capable of suggesting missing elements in incomplete models, aiding developers. It used the Eclipse Modeling Framework (EMF) utilities for extracting Ecore and XMI specifications, complemented by a Java-based custom parser. The cornerstone of the approach was Graph Machine Learning (ML) similarity, which utilised graphical notations for comparing model graphs pairwise. Consequently, the system offers top-ranked recommendations based on graph similarity, improving model completion. This method represents an application of ML in MDE, aiding in identifying dissimilar components and enhancing model quality. This small-scale system, consisting of 1000 lines of code, was developed over a three-year period by a team of four.
- **Project 2: Reflexive Model-Driven Engineering Tool Development**
  This project centered on the creation of MDE tools, constructed using MDE principles in a reflexive approach. The project team undertook a bootstrapping process to accomplish this reflexive MDE application, employing both textual and graphical notations. The initial version of the tool was written in Java and was later used to generate a new version capable of utilizing UML models. This method is reminiscent of the initial bootstrapping carried out in the development of several programming languages, including C. The development process spanned 8 years, with the dedicated efforts of a 10-member team, resulting in a robust system comprising 1 million lines of code.
- **Project 3: Transitioning Defense System Modeling Tools**
  The third project centered around the defense system domain, specifically focusing on the transition from an old modeling tool to a new one. The transition required a complete re-expression of the existing programs, along with changes in the modeling approach for their core components. A team of six individuals successfully developed this system over a span of nine months, using both agile and V-model approaches. Furthermore, graphical notations were utilized to aid in the clarity and comprehensibility of the models
- **Project 4: Mobile Application for Medical Compliance Monitoring**
  This project focused on the creation of a mobile application in the medical field. Utilizing the JetBrains Meta Programming System (MPS), a series of Domain Specific Languages (DSLs) were developed for the purpose of defining medical compliance rules. The mobile application serves as a personal health monitor, offering users recommendations, guidelines for medical care, and suggested actions for managing their health conditions. This project, developed over an eight-year period by a team of four, followed an iterative and incremental development approach. The scope of the project incorporated 200-300 models. The development process employed both textual and graphical notations, with the latter being used in a more limited manner.
- **Project 5: Development of Language for Children to Control Robots**
  This project aimed to develop a user-friendly language to foster children’s involvement with robotics, with a particular focus on crafting a language distinct from yet similar to Lego’s. Unlike Lego’s block-based programming paradigm, this project adopted a different approach to simplify the process and enhance the user interaction experience for children. The project integrated the use of JSON scripts with an existing tool, demonstrating the incorporation of web-based technology to boost functionality and user experience. A team of five worked on this small-scale project over three years, following an iterative and incremental development approach, and used graphical notations to make the system more intuitive for young users.
- **Project 6: Migration of Legacy Code-bases for Cloud Deployment**
  This project revolved around migrating legacy code, predominantly written in traditional languages like COBOL, to modern, cloud-based platforms, specifically Microsoft’s Azure Cloud. A key challenge was dealing with fragments of low-level assembly language code. To overcome this, a compiler was developed to translate the assembler code using textual notations. The project, highlighting the significance of modernization and adaptability in managing code-based assets, was carried out by a two-member team and encompasses 20,000 to 30,000 lines of code.
- **Project 7: Low-Code Solutions for Data Scientists**
  This project, centered on crafting low-code solutions specifically for data scientists, provided streamlined model deployment and operational execution within a user-friendly environment. A key component was a transformation, which converted data scientists’ requirements into operational instructions compatible with cloud infrastructures. Additionally, tools like Xtext editors and Epsilon were utilized in the development environment, and UML graphical notations were used for model creation. This endeavor spanned three years, was developed by a single developer utilizing a Scrum-like method, and resulted in approximately 10,000 lines of code.
- **Project 8: Model Repository for Banking Systems**
  This project aimed at constructing a model repository designed specifically for the banking industry. It involved the creation of a proprietary metamodel, based on the Meta-Object Facility (MOF) framework, which underpinned the management and organization of various models within the repository. This large-scale project was developed over four years by a team of 50 members, employing an agile Kanban method and resulting in 10,000 to 15,000 lines of code. Both graphical and textual notations were utilized for clear representation and effective communication.
- **Project 9: Modelling Workbench for Cyber-Physical System Security Management**
  This project focused on developing a modelling workbench designed to manage the security aspects of cyber-physical systems effectively. The workbench incorporated a system-level design approach, covering the entire development lifecycle. Visual representations based on the metamodel were created to illustrate system designs. These representations, in alignment with the metamodel, enabled users to interact with and query the models for valuable insights. The project was completed over four years by a team of four, following a Scrum-like method and using graphical notations.
- **Project 10: Development of MDE Tool with Custom Model Transformation Language**
  Since 2010, this project has been focused on building an MDE tool for model transformation, not as a full-time endeavor but as an ongoing development. It integrated an improved tree-based editor, a graphical editor for visualizing model manipulation, and a textual syntax to simplify the development of the transformation language. These features catered to different user requirements within the custom language framework. The project, handled by a distributed team of 4-5 members using an iterative and incremental development approach, resulted in approximately 360,000 lines of code.
- **Project 11: Model-Based Testing for Safety-Critical Systems**
  This project employed the Sussel 1 model-based testing methodology for safety-critical systems over an eight-year period, executed by a team of ten members. Instead of modeling the entire system, the emphasis was placed on structuring functions into major and minor components. System models, created using both graphical and textual notations, represented the expected behavior of integrated hardware-software functions. In parallel, a verification company developed models to interface with the system under test, enabling observation during hardware-in-the-loop and integration tests. Furthermore, the project also developed an in-house testing tool, the RT tester, which generated test cases from system-level models, ensuring comprehensive coverage of requirements. The development followed the V model, culminating in 200,000 lines of code.
- **Project 12: UML to Database Transformation**
  This project revolved around the transformation of UML models to overcome criticisms of the unnecessary complexity of UML as a notation. The project aimed to address these limitations by seeking models that could capture a particular slice or view of the system abstractly, without unnecessary complexities and features. This approach is intended to provide a more focused and streamlined representation of the system, enhancing usability and practicality. The project was carried out by at least 10 developers over a time span of 10 years.
- **Project 13: Tool Chain for MDE Adoption in the Automotive Sector**
  This project aimed to develop a comprehensive toolchain specifically designed to accelerate the adoption of MDE practices in the automotive sector. It simplified the modeling process by integrating existing tools into a user-friendly workspace, eliminating the need for separate installations. The tool chain enabled code simulations using exported artifacts, providing valuable insights into system behavior. To support synchronization among artifacts, a textual domain-specific language (DSL) was incorporated, giving domain experts control over the process. The project was developed over four years by a team of four members using an iterative and incremental approach, incorporating some agile practices, resulting in the production of approximately one million lines of code.
- **Project 14: Code Generator for Embedded Systems**
  This project involved the development of a code generation tool for embedded systems, utilizing both textual and graphical notations. Based on the industry-standard ATL model transformation language, the tool incorporated two major model transformations. The first transformation checked the compliance of an ATL model, represented using textual notations, with code generation constraints, generating a report for necessary corrections. The second transformation refined an abstract model, represented using graphical notations, based on a specified platform, applying various patterns for closer alignment with the code implementation. The project was developed by a team of 2-3 members over a span of 10 years.
- **Project 15: Model-Driven Simulations for AI Architectures in Telecoms**
  This project focused on applying Model-Driven Simulations in artificial intelligence architectures within the telecoms sector, specifically utilizing SysML graphical notations. Collaboration with data scientists aimed to integrate MDE methods into existing data science processes and facilitate the transfer of algorithms across projects. The project aimed to enhance the use of AI through model-based ideas and abstractions, enabling effective deployment and reuse of data science algorithms in various contexts. It has been developed over 5-7 years by 5 experienced developers using an agile-like approach.

**Table 1:** Demographic information of participants.

Participant	MDE Experience	Work Type	Application Domain	Role
P1	3 years	Academia	Artificial Intelligence	Developer
P2	30 years	Academia & Industry	MDE tools	Project manager
P3	30 years	Industry	Defense Systems	Advisor
P4	11 years	Industry	Mobile development	DSL designer
P5	12 years	Academia & Industry	Robotics	Usability evaluator
P6	22 years	Academia & Industry	Finance	Developer
P7	15 years	Industry	Data Science	Industry supervisor
P8	13 years	Industry	Finance	Developer
P9	13 years	Academia & Industry	Cyber-physical systems	Developer
P10	24 years	Academia	MDE tools	Developer
P11	20 years	Academia & Industry	Safety-critical systems	Programmer
P12	30 years	Academia	MDE tools	NA
P13	10 years	Academia & Industry	Automotive	Simulation Orchestration Engineer
P14	15 years	Academia	Embedded systems	NA
P15	25 years	Academia	Telecommunication	Supervisor

**Table 2:** Description of projects.

Proj.	Application Domain	Scale	Durat- ion	Dev. Team	Dev. Method	Notations	MDE Tool
PR1	Recommender systems (AI)	1K LOC	3 yrs	4	NA	Graphical	NA
PR2	MDE tool	1M LOC	8 yrs	10	Agile	Graph./Text.	Built-in
PR3	Defense systems	NA	9 mos	6	Agile/V-model	Graphical	Built-in
PR4	Mobile app (Medical domain)	200–300 models	8 yrs	4	Iterative-incremental	Textual (lim. Graph.)	JetBrains
PR5	Robotics	NA	3 yrs	5	Iterative-incremental	Graphical	NA
PR6	Finance/Insurance	25K LOC	NA	2	NA	Textual	Built-in
PR7	Data Science/Low-code	10K LOC	3 yrs	1	Scrum-like	Graphical	NA
PR8	Finance/Banking	10K-15K LOC	4 yrs	50	Kanban	Graph./Text.	MagicDraw
PR9	Cyber-physical systems	NA	4 yrs	3–4	Scrum-like	Graphical	NA
PR10	MDE tool	360K LOC	12 yrs (PT)	4-5	Iterative-incremental	Graphical	Eclipse-based
PR11	Safety-critical systems	200K+ LOC	8 yrs	10	V-model	Graph./Text.	NA
PR12	MDE tool	NA	10 yrs	10+	Agile	Textual	Built-in
PR13	Automotive systems	1M LOC	4 yrs	4	Iterative/ Agile	Textual	Xtext
PR14	Embedded systems	NA	10 yrs (PT)	2–3	NA	Graph./Text.	NA
PR15	Telecom.	NA	5–7 yrs	5	Agile-like	Graphical	Eclipse/ Papyrus

### 4.3 Strengths of model-driven engineering

In order to gain comprehensive insights into the utility and challenges of Model-Driven Engineering (MDE), we asked participants about their personal experiences in selected projects, focusing on what elements of MDE were the most and the least effective. The goal was to identify the strengths of MDE in improving development efficiency and product quality, as well as its potential shortcomings. The following subsections summarize the key findings.

#### 4.3.1 Robustness and reliability.

Among the strengths of MDE identified by the participants were its robustness and reliability (specifically mentioned by 7 out of 15 participants). When followed diligently, MDE processes can lead to high-quality, consistent outcomes—especially in environments with strict quality controls. One participant commented that:

“What worked well is there is reliability... Doing MDE, you modified the model, generate the code... you have to have code reviews and all that kind of thing. So we have a robust process."

Another participant explained how MDE improves quality through consistency and automation:

“The quality is higher usually due to consistency... you only update the model, and then the changes get propagated... That’s much harder to do manually.”

#### 4.3.2 Domain analysis and requirements.

Many participants highlighted how MDE supports deep domain analysis and clear requirements, helping to generate and transform data effectively (8 out of 15 participants).

“I think the part that works best... is this domain analysis and requirements, then we enable for generation or model transformation of data..."

MDE also supports traceability between models and requirements:

“Models help refine requirements, and requirements have to be traceable to models... They’re both an expression of the same set of things.”

Several participants (3 of 15) appreciated the ability to build abstract models tailored to specific domains:

“What worked really well is creating this abstraction and tweaking the abstraction... to different domains.”

“It provided exactly the value that I was trying to bring... to communicate them in a more professional way... engage them and discuss the designs... more professionally."

“It enabled them to make changes quicker, enable them to understand the systems better.”

MDE’s flexibility to evolve languages and support automated refactoring was also valued:

“I think the ability of evolving the language... perform automatic refactoring... reduce development time... generate documentation... extract more value from audits..."

Participants valued MDE’s domain-specific focus:

“A modelling setup where you can talk in terms of your domain of interest... you can ignore all the stuff you don’t want to talk about."

Finally, one participant described how MDE’s benefits became clear over time:

“It took them some time to appreciate that... But once you introduce changes... they slowly begin to understand the value of using model driven engineering.”

#### 4.3.3 System organisation and architecture.

Some participants (3 out of 15) noted the benefit of employing MDE principles to organise systems, which can enhance their structure, facilitate navigation, and speed up their initial setup. The adoption of MDE can result in a clear, understandable, and efficient system architecture. However, it was also emphasised that successful application of MDE requires a clear understanding of the system’s requirements and goals from the outset. Without this clarity, there is a risk of spending significant effort in organising the system in a manner that may not eventually serve the desired objectives optimally. This aspect underlines the importance of precise goal setting and requirement identification in leveraging MDE to its full potential.

“... in the long term, if you organise your system, and according to these principles, then the architecture becomes a lot easier. And it’s easy to get started in the system. The downside is you sort of have to know what you want, because you’re going to spend a lot of time organizing the system in that way, or using the tools getting started with the model driven engineering tools. And if that’s not something that’s important, then you just spent a lot of time on something that’s not important.”

#### 4.3.4 Accelerating the development process.

One significant advantage of MDE highlighted by participants (by 4 out of the 15) is its ability to accelerate development. Automation in MDE reduces manual coding efforts and shortens time to market.

A participant emphasized this impact:

“If you get the right people using it, it’s really, really effective, you can get some real benefits out of it ... we’ve seen huge improvements in efficiency in generating software ... like 30%, decreases in time, delivery to market, big changes in rapid reductions in costs, improvements in quality."

Another noted the speedup due to automation:

“Speed up due to the automation of certain tasks in software engineering.”

One participant described how MDE made an impossible timeline feasible:

“When you really look at if we do it by hand versus if we use the MDE solution that we have, it has massively reduced timescales ... it literally goes from ‘there’s no time in the world to do this’ to ‘yes, this actually can work.’"

Another added:

“And so it’s definitely shorter development times. It’s, well, again, I’m a language guy. So if you have a good domain specific language, then you have a better connection to a domain, which means that quite often your reaction times are also much faster, much shorter.”

Together, these accounts highlight MDE’s strong role in boosting efficiency, reducing time to market, and streamlining software development.

On the other hand, despite its numerous advantages, participants also highlighted areas where Model-Driven Engineering (MDE) did not perform as well as expected. These areas primarily represent challenges and limitations they encountered during their interaction with MDE, ranging from technical difficulties to conceptual and methodological issues.

### 4.4 Weaknesses of model-driven engineering

However, all participants identified one or more flaws in the current state of MDE tools and methods, which hinder its adoption and success.

#### 4.4.1 Learning curve and accessibility.

The learning curve and accessibility of MDE emerged as significant weaknesses with MDE, according to many participants (6 out of 15). They expressed that MDE involves a steep learning curve, making it less accessible to individuals and organizations. Several factors contribute to this steep learning curve:

1. **Paradigm Shift:** MDE requires a shift in how developers approach software development. Traditional programming focuses on writing code, while MDE emphasizes the creation and manipulation of models. This shift in mindset can be challenging for developers accustomed to more traditional approaches. One participant remarked:
  “It’s a moving target... new programming languages, shifting frameworks... software gets more complex... AI-generated code... All these things have to be taken into context when you’re trying to do MDE, so it’s really an obstacle.”
2. **Complexity of Modeling Languages:** MDE relies on modeling languages such as UML (Unified Modeling Language) and DSLs (Domain-Specific Languages), each with its own syntax, semantics, and concepts. Learning these languages and understanding their intricacies can be time-consuming and challenging.
  “One of the biggest problems I have with UML and other modelling languages is they tend to get overly complex... because everybody wanted everything in.”
  Another participant remarked that MDE is inherently complex due to its various levels of abstraction and the need to understand meta-modeling. Additionally, poor explanations or education may increase the perceived complexity and deter adoption:
  “Perceived complexity... most of the time it’s just that it gets explained badly.”
  Another participant added:
  “It’s more complicated than people think; it’s not a silver bullet.”
3. **Lack of Practical Examples and Comprehensive Documentation:** MDE is a relatively young field compared to traditional software engineering. As a result, there may not be as many practical examples, tutorials, or comprehensive documentation available to help newcomers get up to speed.
  “Documentation is difficult... you don’t get much help on StackOverflow... sometimes you have to have 15 different Eclipse versions to get something working... it does not feel like a good, productive development environment.”
4. **Tooling and Infrastructure:** MDE involves the use of specialized modeling tools and infrastructure. Learning how to effectively use these tools — such as model editors, code generators, and transformation engines — can be daunting. Moreover, integrating these tools into existing development workflows and infrastructure requires additional effort and expertise.
  “Another issue is it’s half technological, but the solution is not... big companies want some guarantees that somebody will be there to explain it and help later along the way."
  In addition, the generic nature of MDE tools may not always fit the specific requirements of every project. Sometimes, it is necessary to develop a new language or switch to a different meta-model to express something project-specific.
  “What’s lacking in tooling depends on your project... tools are generic... projects are individual... this is an unbridgeable gap."

#### 4.4.2 Technological limitations.

Most participants (13 out of 15) highlighted technological limitations as another weakness of MDE. They mentioned that certain tools used in MDE may be outdated or not kept up to date with the latest advancements:

“The interfaces that MDE tools provide are very outdated, especially the visual representation."

This lack of tool support — both from commercial and open-source offerings — poses challenges in effectively implementing MDE practices:

“Both commercial and open-source tools are completely dissatisfactory."

Not only is there a lack of support, but also the complexity of the tools makes them even more challenging:

“You’re dealing with inherent complexity, and in your solution, you also introduce some accidental complexity... that’s very hard to track down and get rid of."

Specific tools such as code generators and model checkers are reported to have issues, not meeting expectations in terms of functionality and performance. These limitations can impede the seamless execution and realization of the benefits associated with MDE.

“Modelling is used everywhere, but it’s not so well integrated... integration with other parts is mostly handled manually."

In addition, participants highlighted issues with tool dependency, maintenance, and version updates. They noted how dependency problems can lead to project delays, and how tooling updates can create new challenges.

“We had a problem with the tool... if I didn’t have alternatives, we might have cancelled the project... there are lots of dependencies."

#### 4.4.3 Adoption challenges and organizational resistance.

Most participants (9 out of 15) identified challenges related to the adoption of MDE within organizations. They note that many organizations are deeply rooted in traditional textual requirements practices, making it difficult to introduce the shift towards MDE. The resistance to change can pose obstacles to the successful implementation of MDE methodologies and tools. Participants further emphasize that integrating MDE into an organization can be a long process.

“It’s a big change to an organisation. Organisations are very heavily wedded to textual requirements. Training takes time and cost. In a small organisation, you can do it in a couple of years. In a large one, it can take a decade.”

In addition to institutional resistance, there is also resistance at the individual developer level:

“One issue is resistance from developers. They felt they lost control because there was this additional technology that they didn’t master.”

This resistance might stem from unfamiliarity with MDE, perceived loss of control, or lack of awareness of its benefits.

Another participant added that a lack of in-house MDE expertise is a major barrier:

“The limitation is the lack of competencies in MDE. It’s difficult to find people that understand it and implement the solutions. This is considered a major risk.”

The industry’s slow adoption of MDE may also be due to a gap between academic output and industrial needs:

“A gap between what’s currently offered from an academic perspective and what industry actually needs.”

For example, some noted that tools like Eclipse hinder adoption:

“Eclipse is fundamentally not ready for industrial software development... it becomes a liability. If I have to choose, I’ll skip MDE to avoid Eclipse.”

“Everyone wants to use VS Code or IntelliJ. No one’s using Eclipse. If your aim is to increase adoption of MDE in industry, that’s a dead end.”

One participant described how MDE is often unclear or poorly scoped in practical settings:

“The biggest challenge is: is this an MDE problem? That’s hard to work out. Most companies won’t spend time on that. They might be doing something MDE-like and not realise it — and lose out.”

Some organisations struggle to sustain MDE internally, especially without skilled personnel:

“We didn’t manage to move maintenance to the internal team. It’s very hard to find people with MDE skills, especially JetBrains MPS. When someone left, it took months to find and train a replacement. The company still relies on us as external consultants.”

It is evident that these barriers to the adoption of MDE have been persistent over many years, suggesting that more concerted efforts are required to address them. A more comprehensive approach to education and training, further improvement in tool usability and alignment with industrial needs, better documentation, and stronger community support could help to alleviate these challenges and make MDE more accessible to a broader range of software developers.

#### 4.4.4 Lack of expertise and MDE professionals.

In the domain of Model-Driven Engineering (MDE), a significant challenge is the lack of expertise and the difficulty of finding professionals with MDE skills (4 out of 15 participants identified this as a problem). This shortage of skills can pose formidable challenges in the maintenance and evolution of systems developed using MDE. One participant emphasised the risks associated with this:

“It’s never a good idea to have only one person understand it. If that person is on leave or leaves the company, all your knowledge walks out the door. With MDE, the risk is high. You need multiple people to take knowledge of this stuff — otherwise, you’re just creating legacy you don’t understand in the future.”

This quote underscores the vulnerability of concentrating MDE knowledge in a single individual and highlights the necessity of spreading such specialised knowledge among multiple team members.

Further complicating the situation is the difficulty of finding consultants with expertise in MDE, which can stifle the evolution of MDE systems during the lifespan of a project. Another participant shared their experience:

“And during the life of the project, we sometimes have problems finding the consultants to keep evolving the system because it’s very hard to find people with skills in MDE.”

### 4.5 Key features and capabilities for effective MDE use and tools

This part of our study focused on identifying key features and capabilities essential for effective MDE tools, notations, and processes. We asked participants what elements should be enhanced or introduced to make MDE more powerful, efficient, and user-friendly. Their responses, grounded in practical experience, highlight avenues for future research and can guide the development of next-generation MDE tools. The following subsections present these features and their importance from the participants’ perspectives.

#### 4.5.1 Simplicity and accessibility.

Several participants (9 out of 15) expressed the need for simplicity and ease of use in MDE. They emphasized that beginners should be able to create simple models (the “Hello World” concept) and then gradually add complexity. The learning curve for starting an MDE project should not feel like setting up a full-fledged software system. One participant said:

“Ability to do things in very simple ways... just jump in and do at a very basic level — ‘Hello world’ — and then extend... without having to get into the complexity unless you really want to.”

Another noted:

“If you want to convince industry to use MDE, it has to be a lot simpler to get a basic project running... getting a base MDE project set up feels as hard as doing a full-fledged software project.”

Participants also stressed the importance of well-designed interfaces. A usable interface can reduce complexity and better support domain-specific needs:

“MDE needs to improve its interfaces... protect the user more from the environment... tailor tools per domain — accountants want Excel files, medical want Excel files — we need strategies per domain and taxonomies to address them.”

Participants also stressed the importance of platform flexibility and compatibility:

“Definition of whether it’s cloud-based or supports local applications — providing flexibility for different usage scenarios.”

“Multi-platform compatibility... run on IntelliJ, VS Code, or web-based platforms — making them accessible and usable.”

These views highlight the importance of making MDE tools simpler, more intuitive, and better integrated into the diverse ecosystems developers already use.

#### 4.5.2 Language and representation.

Most participants (13 out of 15) emphasized the central role of language and representation in MDE. One noted the value of integrating formal ontologies and offering varied representations:

“There should be more integration between formal ontology and MDE. Embedding reasoning engines would help. Also, support for different representations—tables, not just drawings—can broaden the user base.”

Another stressed the importance of textual input alongside graphical views:

“Nobody really replaces Java or C++ with visuals. Textual input is important. SysML v2 got it right—textual first, with graphics generated from it. You can skip drawing entirely.”

A participant advocated for more declarative expressions in MDE, supported by tools that handle the processing:

“The more MDE is declarative and tools handle the processing, the better. It reduces effort spent on tool problems. Right now, many systems feel half-finished.”

Another highlighted the need to evolve languages and meta-models without breaking existing models:

“Support for evolution of languages and meta models without breaking models is necessary. Good version control is key.”

Similarly, a participant favored small, focused, textual DSLs over complex general-purpose languages:

“Smaller languages for smaller domains are better. They’re easier to understand, support cheaper tools, and excite newcomers when everything works from day one.”

Lastly, one participant emphasized the value of small, gradual model transformations:

“Transformations should be small steps. Each step should clarify some part of the design before moving on to the next.”

#### 4.5.3 Consistency, maintenance, and reliability across tools.

Most participants (11 out of 15) also stressed the importance of consistent interaction between MDE components. This becomes critical when metamodel changes need to reflect across textual, visual, and code generation layers. One participant stated:

“It’s interaction links between components. If I add an element to the metamodel, I should be notified in the textual syntax that it’s unused. Same for visual syntax. A code generator should highlight where the new elements are used. That would help usability a lot.”

Another highlighted the value of self-checking models and clearer error messages:

“We need self-checking models. In BIA, we had good completeness and consistency checkers. But users often couldn’t understand the language of the errors—it was hard just to understand what went wrong.”

Flexibility and cross-platform support were also emphasized. One participant noted:

“Tools should be multi-platform—not tied to Eclipse, runnable from the command line, and efficient. Code generation is absolutely critical.”

Finally, participants stressed that reliability must be both visible and lasting:

“Demonstrable reliability should be long term. If people invest now, the tech should still run 10, 20, or 30 years later without requiring a full migration.”

#### 4.5.4 Designing at an abstract level.

Some participants (4 out of 15) emphasized the need for MDE to adopt a higher level of abstraction in system design. They envisioned a future where business end-users could directly express system requirements using tailored domain-specific languages (DSLs). One participant explained:

“Model Driven Engineering is the idea that we’ll design software systems with a higher level of abstraction, using appropriate design models... We want something I call DIY programming—the idea that business end users should be able to specify the kinds of systems they need using domain specific languages tailored to express the requirements directly.”

This vision promotes the creation of DSLs that capture the specific vocabulary and rules of each business domain, enabling end-users to articulate their needs without programming knowledge. Such an approach would bridge the gap between technical and business roles, giving end-users a more active voice in system development.

To achieve this, the MDE community must focus on making these languages user-friendly, intuitive, and expressive. If realized, this shift could transform software development into a more collaborative, user-driven process.

### 4.6 Recommendations for MDE improvement

When we asked the participants about their recommendations for improving Model-Driven Engineering (MDE) tools, notations, and operations, several key themes emerged from their responses: Tool flexibility; Compatibility and standardisation; Long-term maintenance support; Integration with existing processes/tools; Improvements in testing for MDE; Simplification of MDE setup; Community projects; User-centered research/development; Web-based tool interfaces, and Improved technology dissemination.

The following quote is typical of the comments received on these aspects:

“it needs to be more intuitive and focused in the way that engineers think. This is partly what SysML 2 is trying to do. But I don’t think it is probably going to succeed in that because it’s still really complicated.”

The following subsections give details of each of these points.

#### 4.6.1 Tool flexibility.

Some participants (5 out of 15) emphasized the importance of adaptable and customizable MDE tools that suit user preferences and project needs. A key request was the ability to switch seamlessly between textual and diagrammatic representations. One participant highlighted the benefit of full textual operation:

“Most of these features, if not all of them, particularly the ability to be fully textual... [are] optional and [should] run on the command line like a programming language.”

This reflects the desire for tools that accommodate varying levels of expertise and enable a programming-like experience through command-line interfaces.

Flexibility also includes interoperability with other development tools and platforms, reducing switching overhead and improving integration across the development lifecycle. Additionally, tools should evolve with technological changes and be extensible.

Another participant explained the tension between generic tools and project-specific needs:

“What’s lacking in tooling depends on your project... the tools are generic by definition, and the projects are individual... you either become an expert and use or build another tool, or you’re bound to the tool. If you’re using a workbench that is Eclipse-bound and targets only Java... and your customer wants Rust or Julia... then your Java tools are just irrelevant.”

Finally, one participant envisioned combining MDE with LLMs to enhance flexibility and accessibility:

“So you would have the leverage of your frameworks... your MDE models, and layered on top of that, a large language model. So, where you can actually just describe the requirements in natural language.”

#### 4.6.2 Compatibility and standardization.

A recurring theme among some participants (5 out of 15) was the need for greater compatibility and standardization across MDE solutions. They cited incompatibilities between tools, modeling languages, data formats, and methodologies as key barriers to broader adoption. One participant noted:

“Another problem that I see is compatibility across MDE solutions ... there is a vendor locking risk for people considering adopting this solution.”

To mitigate this, participants suggested promoting industry-wide standards for modeling languages, data formats, and methodologies. This would reduce vendor lock-in, improve tool interoperability, and give users more freedom to switch tools based on quality and usability.

Another participant emphasized the importance of ensuring backward compatibility as languages and metamodels evolve:

“Supporting evolution of languages and the meta models without breaking existing models ... A good support for version control.”

Standardization would not only enhance integration but also allow tool vendors to compete on usability and reliability than proprietary features.

#### 4.6.3 Long-term maintenance support and backward compatibility.

Some participants (5 out of 15) emphasized the importance of long-term maintenance and backward compatibility in MDE tools, especially given the significant time and resources organizations invest in adopting these technologies. Sustained support—including updates, bug fixes, and adaptability to new standards—is crucial to ensure ongoing value and usability.

One participant highlighted the importance of trust in the longevity of a tool:

“Maintenance support, ensuring people that if they invest now into your technology, then 10 years later, 20 years later, 30 years later, that it will still work.”

Another addressed the ongoing challenge of maintaining MDE models:

“Maintenance is always a problem in software evolution ... your models get worse if you don’t do anything and they get worse if you do something. So their maintenance is a very large concern.”

Participants also stressed backward compatibility—the ability of new versions of a tool to support models and workflows created with older ones—as essential for seamless updates without risking existing work. Without this, users may be forced to stay on outdated tools or invest heavily in migration.

Ultimately, long-term support and backward compatibility are critical to making MDE solutions sustainable, dependable, and user-friendly over time.

#### 4.6.4 Integration with existing processes and tools.

A recurring theme was the need for MDE tools to integrate smoothly into existing development environments and workflows. Some participants (5 out of 15) stressed that MDE should complement, not replace, established processes and general-purpose programming practices.

One participant emphasized this alignment:

“MDE tools should align with existing processes and tools—like using Git with textual models for diffing and merging. If tools fit normal development and solve clear, small problems, they work. If MDE demands changing everything, it’s disruptive.”

Participants also suggested integration with popular IDEs, version control systems, and CI/CD pipelines, enabling MDE to blend into developers’ routines. Another highlighted the importance of supporting mainstream languages:

“Fit it into development with normal general-purpose language—not solve everything with MDE.”

A more modular and flexible approach was also recommended:

“I’d like more pluggable MDE—not a harsh framework that forces everything. If we can say ‘these parts with MDE, others without’ and integrate, that helps industry adoption.”

One participant pointed out why large companies avoid proprietary DSLs:

“[The company I worked in] didn’t want to invent their own DSL—suppliers would demand payment to learn it. [Another company name] had the same concern during model-based testing.”

Ultimately, better integration makes MDE more practical, less intrusive, and easier to adopt within diverse and established software ecosystems.

#### 4.6.5 Improvements in testing.

Some participants (4 out of 15) emphasized the need for stronger testing capabilities in MDE tools, especially for integration testing. Testing is crucial for ensuring tool reliability and user trust.

One participant stated:

“Better testing, better integration tests so you don’t use a tool that will break everything, and backward compatibility so people don’t see you as unreliable.”

This highlights the importance of both robust integration testing and maintaining reliability over time to avoid damaging the tool’s reputation.

Suggestions included incorporating automated test case generation, leveraging models to create input scenarios systematically, and deeper integration of model-based testing (MBT). These techniques can ensure consistency between models and generated code, catching errors early.

Enhancing testing capabilities—through integration tests, automation, and MBT—can boost confidence in MDE tools and support their wider adoption across the software industry.

#### 4.6.6 Simplified core setup.

Several participants (5 out of 15) stressed the need to simplify MDE tools’ core setup and model abstractions, as complexity often hinders adoption—especially for newcomers.

One participant noted:

“Simplify your core setup, model abstractions or work inside the tool, and make sure all your translation programs tie together and don’t generate stuff that raises faults and errors.”

They emphasized streamlining initial setup, improving model abstractions, and ensuring robust translation programs that don’t introduce faults. These improvements would allow designers to focus on core challenges rather than configuration hurdles.

Another participant echoed the need for simplicity across user roles:

“Simplify your model abstractions... Anything that allows the designer to focus on the conceptually hard bits. For end users, it’s about using existing metamodels and transformations without needing deep architectural knowledge.”

In short, simplified setup, clearer abstractions, and reliable transformations would make MDE tools more accessible, helping both expert architects and casual end-users work effectively.

#### 4.6.7 Community projects and collaboration.

Some participants (3 out of 15) highlighted the value of community-driven efforts in advancing MDE, particularly through collaborative development and shared best practices.

One participant emphasized the academic role:

“More community projects, where we, as academia will try to enhance and progress [MDE techniques and applications].”

Another stressed the need for academia to back experiential insights with evidence:

“We need to invest in more experiential research... Many things I discussed today are from my experience in robotics. We need to move effort and resources onto backing up our research.”

A third participant praised the active MDE community and its role in knowledge exchange and innovation:

“There’s a very active community... People think outside the box. For example, in finance, instead of auditing every system, they audited the MDE setup and code generator—so anything it produces is automatically validated. That saves time and builds trust. These insights often come from being in the community and sharing experiences.”

Together, these reflections point to the critical role of collaboration in strengthening research, tools, and real-world applications within MDE.

#### 4.6.8 User-centric research and development.

Several participants (7 out of 15) pointed to a disconnect between MDE research and the practical needs of end users. They advocated for more experimentation with real-world scenarios and a stronger focus on usability and automation.

“MDE processes still lack experimental parts that can be automated—validation with real customers, repeated and reusable experimentation across versions. This is especially critical in domains like cyber-physical systems, autonomous cars, and smart policies, where continuous experimentation is key.”

Another participant suggested the field would progress more effectively by focusing on practical user benefits rather than overly complex features:

“MDE would be further ahead if, instead of focusing on ultra-sophisticated code transformations, it focused on usability—like offering fully textual modeling options and command-line automation.”

These views underscore the need for user-centered development that prioritizes real-world applicability, ease of use, and continuous feedback from end users.

#### 4.6.9 Browser-based tools.

Some participants (4 out of 15) highlighted a growing expectation for MDE tools to be accessible via web browsers, reflecting broader trends in software usability and cloud-based development.

“There is a problem in the fact that it’s not running in the browser, while nowadays many people really expect something like MDE running in the browser.”

Browser-based tools offer several advantages: they eliminate the need for installation, enhance cross-platform access, and support remote collaboration. They also often provide more user-friendly interfaces, which could lower the entry barrier for new users.

“We also need to work on web-based [tools], making some of our tools interfaces into web-based, so that a lot of things are hidden from the user.”

Participants emphasized that tying tools to specific IDEs like Eclipse limits adoption, especially in large organizations that prioritize flexibility and cross-environment compatibility.

“Cloud-based APIs are the preferred way. Anything tied to a particular IDE becomes fundamentally uninteresting... Web-based applications are just the way most companies are moving.”

Despite the benefits, transitioning to browser-based platforms poses challenges such as maintaining performance, ensuring security, and enabling offline capabilities. Nonetheless, the demand for accessible, web-based MDE tools is a strong signal for future development priorities.

#### 4.6.10 Improving dissemination of technology.

Some participants (5 out of 15) emphasized the need to improve how Model-Driven Engineering (MDE) technologies are communicated and shared—both within technical circles and with non-technical stakeholders who influence adoption decisions.

A recurring theme was the importance of simplifying complex ideas and reducing technical jargon to make MDE more accessible. One participant stressed:

“Refining a bit our dissemination of the technology, more collaboration between us as well so that we refine the dissemination of the technology so that there is more awareness.”

Improved dissemination would involve collaborative efforts among researchers, practitioners, and tool developers to produce user-friendly materials such as tutorials, documentation, webinars, and hands-on workshops. These resources should cater to diverse learning styles and technical backgrounds.

Another participant noted the importance of tailoring communication to different user profiles, such as software versus mechanical engineers, and suggested using ontologies to clarify domain-specific terminology:

“Even the mechanical engineer and a software engineer... prefer different representations. They even have problems to argue about the meaning of the vocabulary... So we can use ontologies... instead of just writing a glossary... you can use an ontology where you have a formal representation of that domain.”

The challenge of making MDE approachable was described as a kind of “art of abstraction," where the goal is to present the value of MDE without overwhelming users with technical detail:

“I think that it is a mammoth task on how you present MDE to people... we need to present MDE in an abstracted way so that they get protected from the technical details, and they trust the technology and use it.”

Together, these perspectives underscore the importance of improving how MDE is introduced, taught, and explained—both to accelerate adoption and to build broader trust in its capabilities.

A comprehensive overview of each project’s strengths, weaknesses, key features, and corresponding recommendations is provided in S3 Appendix.

## 5 Discussion

In this section we give an overall summary and analysis of our results with respect to the research questions Q1 and Q2 of Sect 3.1.

Regarding research question Q1.1, *What are the main strengths of MDE and the MDE tools and methods?*, all participants confirmed that MDE had contributed value to their project developments, with the strongest support for the following aspects, ordered by their importance:

1. *Domain analysis and requirements* – that MDE adds rigour to the requirements analysis process, enabling the precise specification of requirements in models at appropriate levels of abstraction, and supporting reuse and requirements tracing.
2. *Robustness and reliability* – that MDE enables the consistent production of high quality software and the management of complex development processes.
3. *Accelerating the development process* – facilitating the automation of code production from models and of other development steps.
4. *System organisation and architecture* – enabling a systematic architecture to be applied to a system.

Overall, the responses to this question show that the benefits claimed for MDE by MDE researchers are genuinely experienced in real-world projects.

Nonetheless, regarding question Q1.2, *What are the main weaknesses or limitations of MDE tools/methods?*, participants consistently highlighted the following issues:

1. *Technological limitations* – especially outdated and excessively complex tools with poor support for system evolution.
2. *Adoption challenges and organisational resistance* – conflicts of MDE with existing development practices, and resistance to change due to the investment that organisations and individuals have in traditional practices.
3. *Learning curve and accessibility* – the disruption of existing practices caused by MDE, language/tool complexity and poor support in terms of documentation and examples.
4. *Lack of expertise and MDE professionals* – the shortage of MDE skills and the consequent risks of adopting MDE.

These factors discouraged the adoption of MDE, increasing the apparent costs and risks for businesses to adopt or to sustain MDE use. Problems of learning curve and skill availability are also cited as issues with MDE in the survey of [1]. The challenges identified in using MDE are one reason why most software practitioners only use MDE in a limited way, if at all, according to the survey of [8].

Regarding research question Q2.1, *What are the key features of MDE tools, methods or notations that should be present?* participants mentioned the following points:

1. *Language and representation* – the use of smaller and more domain-focussed modelling languages, and especially to use textual languages instead of visual (diagrammatic) languages.
2. *Consistency, maintenance and reliability across tools* – the ability to ensure, demonstrate and maintain consistency between different development artefacts and between different tools, for example in the situation where a metamodel changes.
3. *Simplicity and accessibility* – reduce the effort needed to get started in using MDE, and improve the user focus of MDE tools, methods and notations.
4. *Designing at an abstract level* – supporting domain-specific abstractions to enable end-users to participate directly in the development process.

These features would help to reduce the problems identified in responses to Q1.2. In particular, text-based modelling languages should be more acceptable to developers skilled in traditional textual programming languages, and help to accelerate the creation and evolution of models via simpler tooling.

Similar themes emerged in response to Q2.2, *What specific changes/additions are recommended to increase effectiveness of MDE tools/methods/notations?*:

1. *User-centric research and development* – greater pragmatic focus on real-world needs instead of theoretical aspects.
2. *Tool flexibility* – tools should be adaptable and customisable to the needs of specific projects, and to be interoperable with other software tools/platforms.
3. *Simplified core setup* – as with point 3) for Q2.1, participants emphasised the need to simplify the setup of MDE tools and reduce the effort needed to create models.
4. *Compatibility and standardisation* – greater consistency between different MDE tools and languages, including stronger standardisation of these.
5. *Long-term maintenance support* – businesses need to be confident that MDE artefacts will still be usable in the long-term and that there is long-term support for MDE tools.
6. *Integration with existing processes and tools* – seamless integration of MDE tools with mainstream development processes and software languages.
7. *Improvements in testing* – improved support for testing by MDE tools, such as model-based testing capabilities.
8. *Improving dissemination of technology* – greater effort to make MDE tools, methods and benefits more widely understood and accessible.
9. *Browser-based tools* – web-based MDE tool support to facilitate accessibility.
10. *Community projects and collaboration* – published datasets and platforms to improve MDE application.

Improved integration, user-focus and customisability are also emphasised in [1], who call for MDE tools to provide *first-class support for customisation and domain-specific modelling*.

A point mentioned by some participants is the potential future impact on MDE of powerful machine learning tools such as large language models (LLMs): [9,10]. LLMs are being used by developers to generate code directly from natural language requirements without the need to write specifications, however there remain substantial limitations of reliability in this approach due to the fundamentally stochastic nature of LLMs: [11]. Instead, LLMs could be used to address some of the problems identified with MDE by our research, in particular to reduce the effort required to create models, by assisting users to create models from textual requirements. Application of LLMs to requirements formalisation is investigated by [12–14]. The results show that some requirements formalisation ability is present in LLMs such as GPT-4, however specific fine-tuning of a suitable (model-aware) LLM for the formalisation task is necessary in general to improve this capability. Future generations of LLM technology may have sufficient capabilities to provide substantial and reliable assistance for MDE processes and hence reduce the obstacles to MDE use identified by our study participants.

LLMs and other non-symbolic machine learning techniques seem particularly relevant for transforming informal or semiformal natural language requirements into formal artefacts such as software specifications or model transformations, using techniques such as model-transformation by-example (MTBE): [15,16]. However for learning precise mappings between formal artefacts such as formalised specifications and code, symbolic machine learning may be more appropriate than non-symbolic ML, because such techniques can (in principle) learn completely precise and explicit mappings: [17,18].

**Recommendations.** Based on the findings from this study, we propose the following practical recommendations to improve the usability and adoption of MDE tools:

- **For tool developers:** Prioritize flexibility, backward compatibility, browser-based access, and integration with existing development workflows. Enhancing user interfaces and simplifying core setup processes would also significantly lower the adoption barrier. ML support for model creation and other MDE steps can also be used to enhance MDE tool usability.
- **For educators:** Align teaching of MDE concepts with real-world industry practices and concerns. Include hands-on exercises with modern tools, and emphasize the practical benefits of abstraction and domain-specific languages.
- **For organizations:** When considering MDE adoption, assess tool compatibility with existing infrastructure and development processes. Encourage pilot programs and invest in training to bridge the gap between technical and non-technical stakeholders.

These recommendations aim to address the deficiencies and obstacles with MDE use which have been identified by the practitioners in our study.

## 6 Limitations and threats to validity

This section considers limitations of the study and threats to the validity of our results.

### 6.1 Conclusion validity

Conclusion validity refers to the degree to which the conclusions accurately represent the data collected and can be generalized to a broader population. In this study, we interviewed 15 practitioners from both academia and industry to gain insights into model-driven engineering (MDE). The inclusion of participants from diverse backgrounds and working in a wide range of domains (medical, defense, robotics, finance, automotive, etc) enhances the robustness of our findings and supports the validity of our conclusions.

We conducted semi-structured interviews with open-ended questions, allowing for a thorough exploration of the topic and capturing a variety of perspectives. This methodology ensured that responses reflected participants’ genuine experiences with MDE practices.

To minimize potential biases, we selected participants actively engaged in MDE and conducted the interviews online, making it convenient for them to express their thoughts freely. While our sample size is limited to 15 participants, we aimed for variation in professional background and domain experience. Nevertheless,the results should be interpreted cautiously as we do not claim generalizability and encourage future research with larger, randomized samples. Additionally, incorporating complementary research methods, such as focus groups or surveys, could further validate the conclusions and provide a more comprehensive understanding.

### 6.2 Internal validity

Internal validity addresses the extent to which observed effects or relationships can be attributed to the factors under investigation, while minimizing alternative explanations. To ensure internal validity, we designed the study with rigorous data collection procedures and consistent application of a semi-structured interview protocol across all participants. This approach helped minimize variability and interviewer bias.

We took additional measures to reduce bias, such as creating a comfortable environment for participants, encouraging openness, and reducing social desirability bias. By selecting practitioners with varied experiences in MDE from both academia and industry, we sought to capture a broad range of perspectives and to limit the influence of context-specific biases.

Data analysis was conducted using systematic coding and thematic analysis, with multiple researchers reviewing and reaching consensus on themes. This collaborative approach enhanced internal validity by reducing individual biases and ensuring reliability.

Despite these efforts, limitations remain. Participants may have provided socially desirable responses or experienced recall bias, and the researchers’ perspectives could influence the interpretation of the data.

### 6.3 External validity

External validity refers to the extent to which the findings of this study can be generalized to other populations, settings, or contexts. To improve external validity, we included participants from a range of domains and organizations, both in academia and industry. This diverse sample allowed us to capture a wide array of MDE experiences, increasing the applicability of our results to a broader audience of practitioners.

The use of semi-structured interviews with open-ended questions enabled the collection of detailed, personal insights, further reinforcing the relevance of our findings beyond the immediate study context.

However, external validity is shaped by the specific characteristics of the participants and settings. While the diversity in our sample enhances generalizability, the findings may still be context-dependent, requiring cautious application to significantly different environments.

Future studies should consider expanding the participant pool to include more industries and geographic regions. Employing mixed methods, such as surveys or focus groups, would also provide additional perspectives and help to validate the findings across different settings.

### 6.4 Construct validity

Construct validity refers to the extent to which the measures used in a study accurately represent the constructs of interest. In this study, we aimed to ensure construct validity by carefully operationalizing key variables related to MDE through a thorough literature review and a theoretical framework that guided the research questions and interview protocol.

The semi-structured interview format allowed participants to provide detailed responses about their MDE experiences. Open-ended questions helped capture a wide range of relevant constructs, ensuring comprehensive coverage of key aspects of MDE.

We employed multiple coders to analyze the interview data through collaborative coding, reducing individual bias and enhancing the accuracy of the identified themes. Regular discussions between coders ensured consistency in data interpretation.

Nevertheless, construct validity may still be influenced by factors such as the subjective nature of interviews and variations in participants’ interpretations of questions. While our approach was grounded in a solid theoretical framework, it may not fully capture the complexity of MDE perspectives.

To address these limitations, future studies could utilize additional methods, such as surveys or document analysis, to triangulate the constructs and further validate the findings. Feedback from MDE experts would also be beneficial in refining the study’s constructs.

## 7 Related work

In this section, we consider the related empirical studies that have been conducted within the realm of Model-Driven Engineering (MDE). These studies offer valuable insights into the adoption and practical application of MDE in the industry. However, our focus will be on those studies that utilize a specific methodological approach: interview-based research.

In their work, Bucchiarone et al. [2] conducted a series of events in 2017 and 2018, focusing on the evaluation of the current state and evolution of MDE. Gathering experts from diverse sectors, the study identified persistent and newly emerged challenges in the field. The paper offered a reflection on these challenges, categorizing them chronologically based on their time of emergence: foundational, domain-specific, and tool-related. An increased awareness by practitioners of the limitations and difficulties in applying MDE in practice was recognized, mainly due to inflated expectations of MDE tools. The paper also noted the growing relevance of social aspects, including collaborative modeling and confidentiality issues. The authors provided a roadmap for future MDE research, outlining grand challenges that could guide forthcoming initiatives. Our survey identifies many of the same issues as [2], however with a focus on the present-day state of MDE practice rather than trends over time. Subsequent to [2], ML and LLM use has become a significant factor in software engineering, together with an increasing trend towards low-code software development, and these new aspects are reflected in the results of our survey (e.g., projects 1 and 7).

In the study of [19], Hoppner et al. conducted interviews with 56 participants from industry and research sectors to evaluate perceptions of model transformation languages (MTLs) in model-driven development. The study identified that perceptions of MTLs were strongly influenced by the general-purpose expressiveness of general-purpose languages, the domain-specific capabilities of MTLs, and available tooling. Additionally, the choice of MTL, the intended use case, and stakeholder skills were found to moderate these influences. Despite some positive experiences with MTLs, the study concluded that there remains a need for more empirical evidence to enhance language capabilities and tooling, and to improve the overall viability of MTLs. In contrast to our survey, the study of [19] primarily focused on issues with the use of transformation languages, but did not explore the usability aspects of MDE in depth.

In their significant work, Whittle et al. [20] investigated the adoption issues of MDE. They argued that while poor tool support is often pointed to as the primary barrier, social and organizational factors are equally impactful. Through a rigorous analysis of 39 interviews conducted in two separate studies, they developed a taxonomy of technical, social, and organizational considerations related to MDE tool use. They identified that most MDE tools are developed by individuals with a strong technical background but with limited exposure to human-computer interaction or business aspects, often leading to a situation where tools drive users’ thought processes. The authors underscore the need for MDE research to focus on unexplored areas like support for early design stages and creativity in modeling, rather than competing with existing market solutions. They concluded that while MDE can be beneficial, its adoption is often challenging. The authors advocate for the creation of simpler, more adaptable tools that account for a wide range of technical and non-technical factors.

The survey of [20] was extended in [21], where the authors contribute a taxonomy of considerations related to MDE tool use, which can serve as a guide for future tool development. They conclude with recommendations for the MDE community to shift focus towards established Human Computer Interaction (HCI) methods and to explore unaddressed areas of modeling, rather than vying with existing commercial solutions.

Mohagheghi et al. [22] examined the application and adoption of MDE in industry. The study involved four large industrial partners from a European Integrated Project, who were exploring complex systems using MDE techniques. Their findings emphasized that participants found MDE useful for tackling complex system development problems. However, the methodology and tools were generally perceived as challenging to use. These aspects improved with increased MDE usage. The study revealed a demand for mature, technologically advanced tools that can handle intricate models and are easy to integrate with other tools and processes. It was noted that current MDE adopters often invested resources in developing internal tools, demonstrating both the market’s tool shortcomings and the need for domain-specific solutions and tailorable tools.

The studies by Mohagheghi et al. [22] and Whittle et al. [20,21] have made significant contributions to the understanding of MDE and its adoption in academia and industry, and have informed subsequent research and practices. However, these studies are now notably dated, and since then, MDE technologies, tools, practices, and the broader software industry may have significantly evolved. Therefore, while the insights from these studies remain valuable, it is important to interpret them in the context of today’s software development environment. Newer studies or updates to these foundational works would likely provide a more accurate reflection of the current state of MDE adoption and use in the industry. Such studies would benefit from considering the changes and advancements that have occurred in the MDE landscape since the time of the original studies. Our survey helps to provide a more contemporary picture of the state of practice of MDE and the issues associated with MDE use.

## 8 Conclusion

In this paper, we conducted an in-depth examination of the utilisation of Model-Driven Engineering (MDE) through interviews with 15 practitioners across diverse domains. Our aim was to explore the strengths, weaknesses, and key features of MDE tools, methods, and notations, as well as to identify recommendations for enhancing their effectiveness.

The findings from our study offer valuable insights into the practical implications of MDE in real-world projects. Participants acknowledged the significant value that MDE brings to project development, particularly in terms of enhancing robustness, reliability, and accelerating the development process. Moreover, MDE was noted for its contribution to domain analysis, requirements specification, and systematic system organization and architecture.

However, despite the evident benefits, participants also highlighted several challenges and limitations associated with MDE adoption. Issues such as the steep learning curve, technological limitations, organizational resistance, and the shortage of skilled professionals were identified as barriers to widespread adoption.

To address these challenges and enhance the effectiveness of MDE, participants provided insightful recommendations. These include simplifying tool accessibility and language representation, ensuring consistency and maintenance across tools, designing at an abstract level, and fostering tool flexibility and compatibility. Furthermore, suggestions for improvements in MDE testing, integration with existing processes and tools, and increased dissemination of MDE technology were also emphasized. By addressing the identified challenges and incorporating the suggested enhancements, the MDE community can work towards realizing its full potential in software development practices, ultimately fostering innovation and efficiency in the field. As a future work, we plan to conduct user experience (UX) research to understand in detail how practitioners interact with MDE tools and artifacts, identify usability issues, and gather feedback for improving the design and usability of MDE tools.

## Supporting information

S1 Appendix Interview Guide for MDE practitioners. Uploaded as a separate file.
(PDF)

S2 Appendix Information Sheet for Participation. Uploaded as a separate file.
(PDF)

S3 Appendix Summary of Project Strengths, Weaknesses, Features, and Recommendations. Uploaded as a separate file.
(PDF)
