# Supplementary material for: Report from MDE practice: An interview-based evaluation of model-driven engineering uses
Source: PLoS One. 2025 Nov 5;20(11):e0335461. doi: 10.1371/journal.pone.0335461 (PMC12588451; doi:10.1371/journal.pone.0335461)
Supplement: S1 Appendix — Uploaded as a separate file. (PDF) [file pone.0335461.s001.pdf]

S1 Appendix: Interview Guide

Legend: Semi-structured interview questions used to collect information on MDE practices, experiences, and tool usage.

1. How many years of experience do you have with MDE?
2. What is your professional environment? (Academia, Industry, Both, Academic in an industrial setting, etc.)
3. How many projects using MDE have you been involved in?
4. What MDE tools, methods, or notations do you use? For example, textual versus graphical notations?
5. Can you describe a specific project where you used MDE tools or methods?
6. What was your role in the domain? What was the scale of the project, its duration, the team size, and the final outcome?
7. For this project, could you describe the impact of MDE?
8. What are the primary MDE practices that you have used in your work?
9. How effective were tools/notations/methods in practice?
10. What kinds of issues arose with MDE use?
11. What worked best/failed?
12. What were the principal obstacles to using MDE?
13. Are there any specific issues with model transformation or code generation languages that hinder their use and adoption?
14. Have you encountered any other technical or cultural barriers to using MDE practices or tools?
15. How do you measure the success of using MDE in your projects?
16. In your opinion, what are the strengths and weaknesses of MDE tools, methods, and notations?
17. Has MDE assisted in communication between teams or between developers and stakeholders?
18. Has the maintenance of software models been a problem? If so, what actions were taken to overcome this issue?
19. Has integration of MDE techniques with other development techniques been a problem? If so, what actions were taken to overcome this issue?
20. Have you noticed any improvements in the development process, maintainability, or reusability of software products when using MDE?

21. What features or capabilities do you think are important for MDE tools to have in order to be effective?
22. What changes or improvements would you suggest for MDE tools or methods to make them more usable or effective?
23. Can you describe any other positive or negative experiences you've had with modeling languages, tools, or processes?
