# Supplementary material for: Report from MDE practice: An interview-based evaluation of model-driven engineering uses
Source: PLoS One. 2025 Nov 5;20(11):e0335461. doi: 10.1371/journal.pone.0335461 (PMC12588451; doi:10.1371/journal.pone.0335461)
Supplement: S2 Appendix — Uploaded as a separate file. (PDF) [file pone.0335461.s002.pdf]

S2 Appendix: Information Sheet for Participation

Legend: Information sheet provided to participants before the interview, explaining purpose, procedures, and confidentiality of the study.

Title of study:

Investigating the usability of Model Driven Engineering

Invitation Paragraph:

I would like to invite you to participate in this original research project. You should only participate if you want to; choosing not to take part will not disadvantage you in any way. Before you decide whether you want to take part, it is important for you to understand why the research is being done and what your participation will involve. Please take time to read the following information carefully and discuss it with others if you wish. Ask me if there is anything that is not clear or if you would like more information.

What is the purpose of the study?

The aim of this study is "Investigating the Usability Aspects of Model Driven Engineering", which involves conducting interviews with MDE practitioners in industry and academia regarding the usability of MDE notations, tools, and processes. Specifically, I am interested in finding out about the issues and challenges faced in MDE, and how they can be improved. Additionally, I would like to investigate the experiences of applying MDE, its advantages, shortcomings, and any areas that require improvement. To achieve our aim, this study will involve an interview session with you.

Why have I been invited to take part?

I am inviting people who have been involved in MDE field in order to gain some realistic information regarding usability aspects in model driven development based upon their personal experience and point of view.

Do I have to take part?

Participation is voluntary. You do not have to take part.

What will happen to me if I take part?

If you decide to take part you will be given this information sheet to keep and will be asked to sign a consent form. With your consent, I will arrange to interview you in a quiet area (for confidentiality reasons) on organisation premises, where you work (or at a suitable venue in a local public site if you prefer). We can alternatively use other telecommunication method of your preference (Skype or telephone, etc.). The interview will take approximately 30-45 mins, but it is designed to be flexible so as to meet your needs. The interview will be recorded, subject to your permission. Recordings of interviews will be deleted after transcription. Even if you have decided to take part, you are still free to cease your participation at any time and to have research data/information relating to you withdrawn without giving any reason up to the point of publication in 01/09/2023. The participant will have one-week time after the actual interview to

withdraw, since afterwards data will be encrypted and will no longer be identifiable.

What are the possible benefits and risks of taking part?

There are no foreseeable risks in participating in the study. The main inconvenience to taking part in the study may be that you will be donating around an hour of your time.

Will my taking part be kept confidential?

What is said in the interview is regarded as strictly confidential and will be held securely until the research is finished. Your participation is entirely voluntary. If you change your mind, you are free to stop your participation at any time and to have your data withdrawn without giving any reason up to the point of publication in 01/09/2023. All data for analysis will be anonymised. In reporting on the research findings, I will not reveal the names of any participants or the organisation where you work. At all times there will be no possibility of you as individuals being linked with the data. The UK Data Protection Act 1998 will apply to all information gathered within the interviews and held on password-locked computer files. All information related to this study are in locked cabinets within the premises of King's College London. No data will be accessed by anyone other than the research team; and anonymity of the material will be protected by using false names.

What will happen to the results of the study?

The result of the study will be used anonymously for papers (publications).

Who should I contact for further information?

If you have any questions or require more information about this study, please contact me using the following contact details:

Email: Hessa.1.alfraihi@kcl.ac.uk

What if I have further questions, or if something goes wrong?

If this study has harmed you in any way or if you wish to make a complaint about the conduct of the study you can contact King's College London using the details below for further advice and information:

Name: Kevin Lano

Email: kevin.lano@kcl.ac.uk

Telephone: +44 020 7848 2832

Office: (N)6.01, Bush House, Strand Campus

Thank you for reading this information sheet and for considering taking part in this research.
