# Supplementary material for: Report from MDE practice: An interview-based evaluation of model-driven engineering uses
Source: PLoS One. 2025 Nov 5;20(11):e0335461. doi: 10.1371/journal.pone.0335461 (PMC12588451; doi:10.1371/journal.pone.0335461)
Supplement: S3 Appendix — Uploaded as a separate file. (PDF) [file pone.0335461.s003.pdf]

S3 Table: Summary of Project Strengths, Weaknesses, Features, and Recommendations

Legend: Summary of project strengths, weaknesses, features, and recommendations for all projects analyzed in the study.

Table 1: Summary of Project Strengths, Weaknesses, Features, and Recommendations

Proj.	Key Strengths	Key Weaknesses	Key Features	Recommendations
PR1	Accelerate development process	Technological limitations, learning curve, accessibility	Simplicity and accessibility, Language and Representation, Designing at an Abstract Level	Tool Flexibility, User-Centric R&D
PR2	Robustness and reliability, Domain analysis and requirements	Technological limitations, adoption challenges and resistance	Simplicity and accessibility, Designing at an Abstract Level, Maintenance and Reliability, Language and Representation	Tool Flexibility, Simplified Setup, Compatibility & Standardization, Community Collaboration, User-Centric R&D, Improving Dissemination of Technology
PR3	Accelerate development, domain analysis, quality improvement	Adoption challenges and resistance	Language and Representation	Simplified Setup, User-Centric R&D
PR4	Accelerate development, quality improvement, robustness	Lack of expertise, adoption challenges	Maintenance and Reliability, Language and Representation	Browser-Based Tools, Compatibility & Standardization, Long-Term Maintenance Support, Improving Dissemination of Technology
PR5	Accelerate development	Technological limitations	Consistency, Language and Representation, Maintenance and Reliability	Improvements in Testing, Integration with Existing Processes and Tools

Continued on next page

Table 1 Continued from previous page

Proj.	Key Strengths	Key Weaknesses	Key Features	Recommendations
PR6	Domain analysis, system organization	Technological limitations, learning curve, lack of expertise	Maintenance and Reliability	Long-Term Maintenance Support, Improving Dissemination of Technology
PR7	Domain analysis and requirements	Technological limitations, lack of expertise	Simplicity and accessibility, Maintenance and Reliability	Tool Flexibility, Compatibility & Standardization
PR8	Domain analysis, robustness and reliability	Technological limitations, learning curve, adoption challenges	Simplicity and accessibility, Language and Representation, Maintenance and Reliability	Compatibility & Standardization, Integration with Existing Processes,Browser-Based Tools, User-Centric R&D
PR9	Robustness and reliability, maintainability improvement	Technological limitations, adoption challenges, learning curve	Language and Representation, Maintenance and Reliability	Compatibility & Standardization
PR10	Domain analysis, productivity improvement	Technological limitations	Simplicity and accessibility, Maintenance and Reliability, Language and Representation	Integration with Existing Processes and Tools, Simplified Setup, User-Centric R&D
PR11	Robustness and reliability, domain analysis	Technological limitations, adoption challenges, learning curve	Simplicity and accessibility, Language and Representation, Maintenance and Reliability	Improvements in Testing, Integration with Existing Processes,Simplified Setup, Tool Flexibility
PR12	Domain analysis, system organization	Technological limitations	Designing at an Abstract Level, Maintenance and Reliability, Language and Representation, Consistency	Simplified Setup, Improvements in Testing, Tool Flexibility, Long-Term Maintenance Support, User-Centric R&D

Continued on next page

Table 1 Continued from previous page

Proj.	Key Strengths	Key Weaknesses	Key Features	Recommendations
PR13	System organization and structure	Technological limitations, adoption challenges	Maintenance and Reliability, Consistency, Language and Representation, Simplicity	Improvements in Testing, Long-Term Support, Integrating with Existing Processes
PR14	Robustness and reliability	Technological limitations, learning curve, adoption challenges	Language and Representation, Simplicity and accessibility	Community Collaboration, Browser-Based Tools, Long-Term Support, Improving Dissemination
PR15	Robustness and reliability	Technological limitations, adoption challenges, lack of expertise	Simplicity and accessibility, Language and Representation, Designing at an Abstract Level	Community Collaboration, Improving Dissemination, Browser-Based Tools, User-Centric R&D
